# Benzonate derivatives of acetophenone as potent *α*-glucosidase inhibitors: synthesis, structure–activity relationship and mechanism

**DOI:** 10.1080/14756366.2019.1604519

**Published:** 2019-05-09

**Authors:** Wen-Jia Dan, Qiang Zhang, Fan Zhang, Wei-Wei Wang, Jin-Ming Gao

**Affiliations:** Shaanxi Key Laboratory of Natural Products and Chemical Biology, College of Chemistry and Pharmacy, Northwest A&F University, Yangling, Shaanxi, China

**Keywords:** α-Glucosidase, acetophenone derivatives, inhibiting mechanism, *in vitro*, *in vivo*

## Abstract

In this article, 23 compounds (**6** and **7a**–**7v**) were prepared and evaluated for their *in vitro α*-glucosidase inhibitory activity. The compounds **7d**, **7f**, **7i**, **7n**, **7o**, **7r**, **7s**, **7u,** and **7v** displayed the *α*-glucosidase inhibition activity with IC_50_ values ranging from 1.68 to 7.88 µM. Among all tested compounds, **7u** was found to be the most efficient, being 32-fold more active than the standard drug acarbose, which significantly attenuated postprandial blood glucose in mice. In addition, the compound **7u** also induced the fluorescence quenching and conformational changes of enzyme, by forming *α*-glucosidase–**7u** complex in a mixed inhibition type. The thermodynamic constants recognised the interaction between **7u** and *α*-glucosidase and was an enthalpy-driven spontaneous exothermic reaction. The synchronous fluorescence and CD spectra also indicate that the compound **7u** changed the enzyme conformation. The findings identify the binding interactions between new ligands and *α*-glucosidase and reveal the compound **7u** as a potent *α*-glucosidase inhibitor.

## 1. Introduction

Diabetes mellitus is one of the major chronic diseases^1^. The incidence and prevalence of diabetes have risen sharply in recent years, and 642 million people might be suffering from diabetes until 2040^2^^,^^3^. *α*-Glucosidase is a type of glycoside hydrolase, which favors in absorption of carbohydrates. By inhibiting its activity, the absorption of small intestine carbohydrates could be delayed, which bring on the reduction of postprandial blood glucose^4^^,^^5^. Moreover, the inhibition of *α*-glucosidase also has positive effects to treat some diseases such as virosis, cancer, and chronic heart failure etc^6^^,^^7^. Nowadays, the *α*-glucosidase inhibitors have been recognised as an efficient therapy in the treatment of type-II diabetes (T2D)^8^. They inhibit membrane bound *α*-glucosidase in the cells lining the small intestine, which in turn decreases the digestion of starch and additional dietary sugars, helping to avoid hyperglycemia and maintain normal blood sugar levels^9^. Therefore, it is significant to discover new classes of *α*-glucosidase inhibitors and to investigate the action mechanism of these inhibitors.

**Figure 1.**
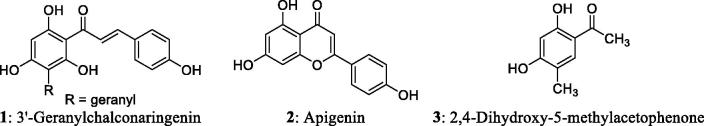
The structures of 3′-geranylchalconaringenin **1**, apigenin **2** and 2,4-dihydroxy-5-methylacetophenone **3**.

Numerous medical and nutritional researches have revealed that the natural phenols took a key position in prevention and control of various diseases like diabetes, cancer, and neurodegenerative^10^^,^^11^. Various natural products have been reported for their *α*-glucosidase inhibition activity. The study of Sun et al.^12^ and Zeng et al.^13^, shows that the natural phenols 3′-geranylchalconaringenin **1** and apigenin **2** (Figure 1) both exhibited superior inhibition activity against *α*-glucosidase than acarbose (a standard drug). The compound 2,4-dihydroxy-5-methylacetophenone **3** was isolated from the cultures of *Polyporus picipes* (Figure 1) by our group as a good fungicide^14–19^, which also showed considerable inhibitory activity against *α*-glucosidase in prescreen. Considering the structural characteristics of existing phenolic *α*-glucosidase inhibitors and the advantages of natural product resources, in this research work about 23 derivatives of 2,4-dihydroxy-5-methylacetophenone **3** were synthesised. The newly prepared analogs were also explored for their inhibitory activity and mechanism against *α*-glucosidase.

## 2. Materials and methods

### 2.1. General

^1^H NMR and ^13 ^C NMR spectra were carried out using a 500 MHz Avance spectrometer (Bruker, USA) or a Varian Mercury 400 MHz spectrometer. Chemical shifts were measured relative to the residual solvent peaks of CDCl_3_ with tetramethylsilane as the internal standard. High-resolution mass spectra were recorded on an AB SCIEX Triple TOF 5600+ spectrometer. The *in vitro* inhibitory activity and kinetic assay of *α*-glucosidase were measured by a microplate reader (Synergy HTX, BioTek Instruments Inc., Winooski, VT, USA). Fluorescence spectra were conducted on a spectrofluorometer (LS55, Perkin Elmer Inc., Waltham, MA, USA). All of the CD spectra were measured at 298 K with a circular spectrometer (Chirascan, Applied Photophysics Ltd., UK). Titration microcalorimetry measurement conducted on a Nano-ITC SV instrument (TA Instruments Ltd., UK). The surgeries were performed under anesthesia and all efforts were made to minimise animal suffering. The blood glucose levels were determined *via* a glucose detection kit (Robio Co., Ltd., Shanghai, China). The jejuna for everted sleeve assays were obtained from adult male Sprague–Dawley rats (SCXK (Shaan) 2017–003, 7 weeks old). The concentrations of glucose in the soaking solution and intestinal sleeve were detected using S-10 biosensor analyzer (Sieman Technology Co., Ltd., Shenzhen, China). All reaction progress was monitored by thin-layer chromatography (TLC) on silica gel GF_254_ (Qingdao Haiyang Chemical Co., Ltd., Qingdao, China) with ultraviolet detection. Reagents were purchased from commercial sources (Bodi Chemical Co., Ltd., Tianjin, China; Aladdin Industrial Co., Ltd., Shanghai, China), and used as received. *α*-Glucosidase (EC 3.2.1.20, Sigma-Aldrich Chemical, St. Louis, MO, USA); 4-nitrophenyl-*α*-D-glucopyranoside (*p*NPG) (99%, Macklin Biochemical Co., Ltd, Shanghai, China).

### 2.2. Chemistry

**Scheme 1.**
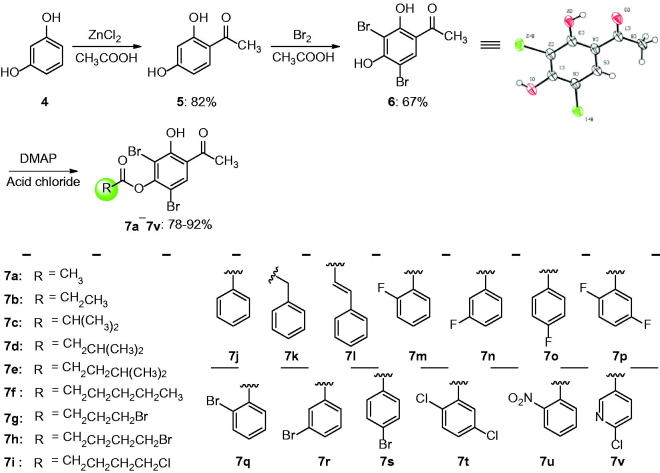
Synthesis of compounds **7a**−**7v**.

The synthesis of natural product **3** has been reported by our group (Scheme S1)^15^. Compounds **6** and **7a**–**7v** were carried out as illustrated in Scheme 1. Details are described below.

#### 2.2.1. Synthesis of 3,5-dibromo-2,4-dihydroxyacetophenone (6)

To a solution of 2,4-dihydroxyacetophenone **5** (1.52 g, 10 mmol) in acetic acid (80%, 20 ml), 3.2 g of bromine (1.04 ml, 20 mmol) dissolved in 10 ml acetic acid was added dropwise with continuous stirring^20^. The mixture was stirred for 15 min. Then the pale red crystals obtained, which were filtered off and recrystallised from benzene to give compound **6** as white crystals, yield (2.06 g, 67%), m.p. 174 − 175 °C. ^1^H NMR (500 MHz, CDCl_3_) *δ* 13.31 (s, 1 H), 7.88 (s, 1 H), 2.60 (s, 3 H); ^13 ^C NMR (125 MHz, CDCl_3_) *δ* 202.0, 160.4, 155.6, 133.4, 115.3, 99.4, 98.9, 26.3. HR-ESI-MS m/z Calcd. for C_8_H_6_Br_2_O_3_ [M–H]^–^ 306.8611, found 306.8610.

#### 2.2.2. Synthesis of compounds 7a–7v

A solution of **6** (1 mmol), 4-dimethylaminopyridine (2 mmol) and acid chloride (1.2 mmol) in dry acetonitrile (15 ml) was stirred at room temperature. The reaction was monitored by TLC. On completion, the resulting mixture was directly evaporated. Then the residue was purified by silica gel column chromatography with petroleum ether and ethyl acetate (10:1, v/v) to produce **7a**–**7v** in 78–92% yield.

### 2.3. Biological evaluation

#### 2.3.1. α-Glucosidase inhibitory assay

The *α*-glucosidase inhibitory assay was performed according to a slightly modified method previously reported^21^^,^^22^. In brief, a series of reaction solutions, including a fixed amount of *α*-glucosidase (10 unit/mL 3.75 µL), different concentrations of inhibitors (the tested compounds or positive controls, 37.5 µL), and sodium phosphate buffer (PBS, 596.75 µL, 0.1 M, pH 6.8), were incubated at 37 °C for 10 min. To initiate the reaction, 112.5 µL of *p*NPG (6.0 mM, as a substrate) was added into the pre-incubated mixtures, and the final volume of reaction system was kept at 750 µL. The *p*-nitrophenol released from *p*NPG substrate was used as the target substance to quantify the enzymatic activity. The absorbance of *p*-nitrophenol was monitored at 405 nm after incubation at 37 °C for 30 min. All samples were analyzed in triplicate, acarbose, and genistein were used as positive controls. The negative control was prepared by adding PBS instead of *α*-glucosidase, the blank was prepared by adding solvent instead of tested compounds, and the inhibition rate was calculated as the following Equation (1).

(1) $$\frac{\left(OD_{control}-OD_{control\;blank}\right)-\left(OD_{test}-OD_{test\;blank}\right)}{OD_{control}-OD_{control\;blank}}\times 100\%$$

Equation (1). Inhibition rate calculation formula.

#### 2.3.2. Sucrose–loading test

The carbohydrate loading test was conducted according as Kato et al.^23^ and Han et al.^24^ reported, with slight modifications. Fifteen male Kunming mice (35–40 g) after an overnight fast were randomly divided into three groups (five mice per group). Sucrose (2.5 g/kg body weight), as well as the tested compound **7u** (20 mg/kg body weight) and acarbose (20 mg/kg body weight) were dissolved in 0.5% sodium carboxymethyl cellulose (CMC–Na) solution, then administered to mice *via* a gavage method, respectively. A blank control group was loaded with 0.5% CMC–Na solution only. The blood samples were collected from the mice’s tail vein at 0, 15, 30, 60, 90, and 120 min, and the blood glucose levels were determined by a glucose detection kit.

#### 2.3.3. Everted sleeve assays

The inhibitory activity of compound **7u** against *α*-glucosidase was assayed on everted intestinal sleeves following the methods of Han et al.^24^ and Scow et al.^25^ with little modifications. After a 12-h fasting, the jejunum of 200–220 g Sprague-Dawley mice was obtained. Then the proximal jejunum was flushed with pre-cold isotonic saline (4 °C) and cut into 3 cm-long segments. Each segment was weighed separately, everted and both ends were secured by tying knots. Then the sleeves were incubated (90 min at 37 °C) in mammalian Ringer’s solution containing sucrose (1.5 mM) and an appropriate concentration of the inhibitor (0.17 mM). Following the incubation, the concentrations of glucose in the soaking solution and intestinal sleeves were tested with biosensor analyzer (S-10). The total glucose concentration was calculated as glucose per gram of gut weight (mmol/g).

#### 2.3.4. Kinetic analysis

To further explore the inhibitory characteristic of this type of compounds, kinetic studies of the selected compound **7u** were performed^3^^,^^13^^,^^26^. Different final concentrations (0.5, 1, 2, 4, and 8 µM) of the inhibitor **7u** around the IC_50_ values were chosen. The concentration of *α*-glucosidase was kept at 1 µM, and the *p*NPG concentrations varied from 2.0 to 12.0 mM in the absence and presence of compound **7u**. The type of inhibition was analyzed on the basis of the Michaelis–Menten constant (*K*_m_) and maximal rate (*V*_max_) of the enzyme. The parameters were determined *via* Lineweaver–Burk plots, which was obtained by plotting enzyme reaction velocity (*ν*) and substrate (*p*NPG), namely, 1/*ν versus* 1/[*p*NPG]. The secondary plot slope against [**7u**] was also determined. The protein concentration was determined according to the Bradford method^27^ using bovine serum albumin as the standard^28^.

#### 2.3.5. Fluorescence spectra measurements

The steady state fluorescence emission spectra, were recorded between 300 and 500 nm at the excitation wavelength of 280 nm at three different temperatures (25, 31 and 37 °C), and both the excitation and emission slits were set at 2.5 nm. Compound **7u** at different concentrations (from 0 to 6 µM) were added into the 3.0 ml solution containing a fixed amount of *α*-glucosidase (2*μ*M). All of the mixtures were held for 5 min to equilibrate before measurements. The fluorescence spectra of buffer were subtracted as the background fluorescence^29–32^.

The synchronous fluorescence spectra of *α*-glucosidase in the absence and presence of compound **7u** were scanned by setting the excitation and emission wavelength intervals (Δ*λ*) at 15 and 60 nm, respectively. To eradicate the probability of re-absorption and inner filter effects in UV absorption, all of the fluorescence data were corrected for absorption of exciting light and emitted light, based on the Equation S1^3^^,^^13^.

#### 2.3.6. Titration micro-calorimetry measurement

Titration of the compound **7u** into the *α*-glucosidase was performed at 25 °C. All the solutions were prepared in 0.1 M PBS (pH 6.8). The *α*-glucosidase solution (2.5*μ*M) was placed in the 1.3 ml sample cell of the calorimeter, and the injection syringe was loaded with the solution of **7u** (30*μ*M, in 0.05% DMSO). The solution of compound **7u** was titrated into the sample cell at 25 °C as a progression of 25 injections of 10*μ*L. The time delay between each two injections was 300 s. To ensure comprehensive mixing, all the sample was stirred at a speed of 300 rpm during experiment. The control experiments included the titration of the compound **7u** solution into 0.1 M PBS (pH 6.8)^33–35^.

#### 2.3.7. Circular dichroism measurement

The CD measurements of *α*-glucosidase in the presence of compound **7u** were conducted in far–UV region (190–250 nm), under nitrogen environment using a 1.0 mm path length cuvette. The concentration of *α*-glucosidase was maintained constant (2 µM) while the **7u** concentration was varied as 0, 1, 2, and 4 µM. All observed CD spectra were recorded in 0.1 M PBS (pH 6.8) at 25 °C^13^^,^^31^.

### 2.4. Statistical analysis

All data are expressed as mean ± SD. The statistical analysis was performed with GraphPad Prism 7.0 (GraphPad, La Jolla, CA, USA), using one-way ANOVA followed by the Tukey *post hoc* test (for comparison among three or more groups). The statistical significance was considered at *p < 0.05, **p < 0.01, and ***p < 0.001.

## 3. Results and discussion

### 3.1. Chemistry

The natural product **3** can be easily obtained by reduction and Friedel-Crafts reactions of industrial raw material 2,4-dihydroxybenzaldehyde^15^. The compound **5**, similar to the natural product **3**, can be obtained in good yield (82%) from resorcinol **4** with previously reported catalyst ZnCl_2_–CH_3_COOH (Scheme 1). In order to increase the metabolic stability^36^, the Br was used to replace the hydrogen atoms by conventional halogenation reaction and the moderate yield (67%) was acquired^20^. The structure of compound **6** was also verified by X-ray single crystal diffraction (CCDC 1869203). With this compound **6** in hand, the main focus was on modifying phenolic hydroxyl at OH-4′, which is more reactive than OH-2′ due to presence of intramolecular hydrogen bond. That’s why a series of ester derivatives **7a**–**7v** (78–92% yields) was synthesised, with various chain alkanes and substituted phenyl groups, which in turn varied in chain length, electron-inducing ability and substitution position. All these compounds were thoroughly characterised (see Supporting Information for details, Figures S2–S24). This diversity of product may help to explore the interactions between small molecules and biotarget.

### 3.2. Biological evaluation

#### 3.2.1. *In vitro* α-glucosidase inhibitory activity and SARs

As presented in Table 1, all the compounds were tested for their inhibition rate under 20 µM. Then the active compounds whose inhibition rates were over 70% were evaluated for their IC_50_ values. The IC_50_ values were found between 1.68 and 7.88 μM for **7d**, **7f**, **7i**, **7n**, **7o**, **7r**, **7s**, **7u,** and **7v**, while that of acarbose and genistein was 54.74 and 22.64 μM, respectively. These results indicated that those compounds were more effective against *α*-glucosidase than the standard drug acarbose.

**Table 1.** *α*-Glucosidase inhibition of high active compounds *in vitro*.

Compd.	IC_50_ (µM)	Compd.	IC_50_ (µM)
**3**	16.22 ± 0.41	**7l**	12.07 ± 0.22
**6**	17.38 ± 0.55	**7n**	3.46 ± 0.26
**7c**	19.35 ± 0.46	**7o**	1.97 ± 0.28
**7d**	6.08 ± 0.20	**7r**	4.62 ± 0.24
**7e**	10.89 ± 0.07	**7s**	7.88 ± 0.17
**7f**	6.73 ± 0.81	**7u**	1.68 ± 0.05
**7g**	12.43 ± 0.27	**7v**	1.85 ± 0.27
**7i**	3.27 ± 0.39	**Genistein**	22.64 ± 3.03
**7j**	17.49 ± 0.53	**Acarbose**	54.74 ± 0.16

The SARs of these derivatives were summarised briefly as following: (1) longer straight-chain alkyl at ester substitution sites could increase the level of *α*-glucosidase inhibition, as IC_50_ of compounds **7a** and **7f** were >20 and 6.73 µM, respectively; (2) the introduction of branched alkyl chain are favorable, as inhibiting activity of compounds **7c** (IC_50_ = 19.35 µM) was superior than **7a**; (3) the haloalkyl chain might improve the inhibition, such as compound **7i** (IC_50_ = 3.27 µM). However, compound **7 h** was a special case which lost the activity; (4) the aromatic ring substituted groups were beneficial for improving the activity, except for the compounds **7k**, **7 m**, **7q**, and **7t**. Especially, the large steric and electron-withdrawing groups on the aromatic rings were essential for enhancing the *α*-glucosidase inhibitory activity (the IC_50_ of **7u** was 1.68 µM). Compound **7u** was found as the most high active against *α*-glucosidase among all newly synthesised compounds. We thus furtherly explored its mechanism on *α*-glucosidase inhibition by several methods.

#### 3.2.2. Sucrose-loading test

**Figure 2.**
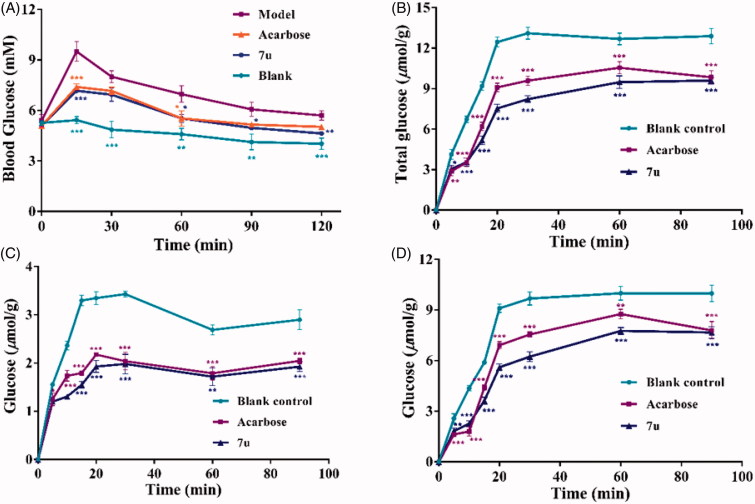
(A) Effects of compound **7u** and acarbose on postprandial blood glucose levels. (B) Inhibitory activity of compound **7u** (0.17 mM) and acarbose (0.17 mM) on mice intestinal *α*-glucosidase. Total concentration of glucose (*μ*mol/g); (C) and (D) Plots of the concentrations of glucose (*μ*mol/g) in and out of the intestinal sleeves, respectively.

The sucrose loading assay was performed to confirm whether compound **7u** kept reduction effects on postprandial blood glucose levels *in vivo*. The blood glucose levels were measured as scheduled time, by the glucose detection kit. As shown in Figure 2(A), the blood glucose level of fasted mice was rapidly increased from 5.40 to 9.50 mM after administration of sucrose (2.5 g/kg body weight) for 15 min. In comparison, the blood glucose level of blank group exhibited almost no variations through the test time course. Meanwhile, an obvious repressive effect on blood glucose concentrations at peaked 15 min was observed for treated group (compound **7u**), which were similar to acarbose. Interestingly, both blood glucose concentrations of **7u** treated group and acarbose treated group were at lower level than the model group, and exhibited no significant difference up to 90 min. It can be inferred that the hyperglycemic inhibition by **7u** was probably associated with its inhibitory activity on the carbohydrate-digestive enzymes. For example, *α*-glucosidase, or the glucose transporters, including sodium-dependent glucose cotransporters (SGLTs) and the facilitative glucose transporters (GLUTs)^24^.

#### 3.2.3. Everted sleeve assays

The *α*-glucosidase is one of the most important carbohydrate digestive enzymes which are found on the surface membrane of the intestine. In order to explore the inhibitory activity of compound **7u** against intestine *α*-glucosidase, the inhibitory activity of *α*-glucosidase on everted intestinal sleeves was tested. As shown in Figure 2(B), both **7u** (0.17 mM) and acarbose (0.17 mM) led to decreasing the concentration of total glucose from the first (5 min) to the last (90 min) time-point, and the concentrations of glucose were found much lower than that of the blank. Additionally, the inhibitory activity of compound **7u** was better than that of acarbose. The glucose concentrations of compound **7u** and acarbose-treated groups in the intestinal sleeves were lower than that of blank (Figure 2(C)), which indicated both **7u** and acarbose could inhibit the activity of glucose transporters, such as SGLT1 and GLUT2, and help reducing the glucose absorption from the intestinal tract to the blood^25^. The compound **7u** also decreased the glucose concentrations in the solution present outside the intestinal sleeves (Figure 2(D)), which suggested that **7u** was more efficient to inhibit the transformation of sucrose to glucose. Accordingly, compound **7u** was taken as a significant inhibitor of *α*-glucosidase. The inhibitory activity of *α*-glucosidase could effectively reduce postprandial blood glucose, which agreed with the result of sucrose-loading test. Hence, the major concentration was exploring the inhibitory mechanism of compound **7u** against *α*-glucosidase.

#### 3.2.4. Kinetic analysis of inhibitory type

**Figure 3.**
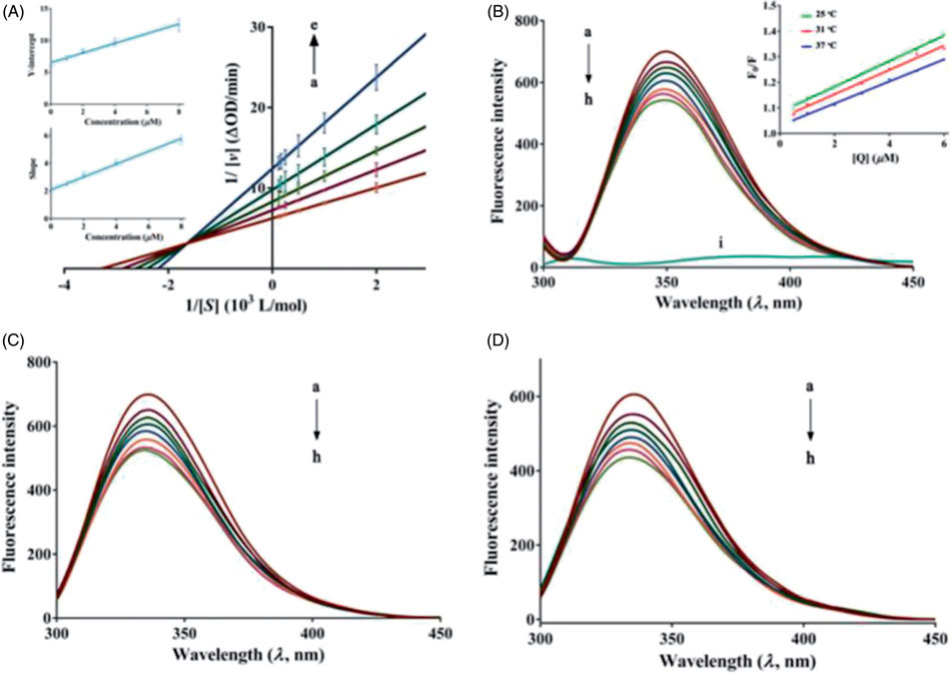
(A) Lineweaver–Burk plots. c(**7u**) = 0.5, 1, 2, 4 and 8 μM for curves a → e, respectively. The inset was the secondary plots of slope (the upper left) and Y-intercept (the lower left) versus [**7u**]. (B) Fluorescence spectra of *α*-glucosidase in the presence of **7 u** at various concentrations (pH 6.8, *T* = 25 °C, *λ*_ex_ = 280 nm, *λ*_em_ = 346 nm). c(*α*-glucosidase) = 2 µM, and c(**7u**) = 0, 0.5, 1, 2, 3, 4, 5 and 6 µM for curves a → h, respectively. Curve i shows the emission spectrum of **7 u** at the concentration of 6 µM. The Stern–Volmer plots for the fluorescence quenching of *α*-glucosidase by **7 u** at different temperatures were inserted. (C) *T* = 31 °C. (D) *T* = 37 °C.

To explore the type of inhibition characterised by **7u** against *α*-glucosidase, the kinetic assay was carried out by Lineweaver–Burk equation and Michaelis–Menten equation (Equations S2–S4). As shown in Lineweaver–Burk double-reciprocal plot (Figure 3(A)), the lines intersected in second quadrant, while, *V*_max_ and *K*_m_ changed with varying the concentration of compound **7u**. These results indicated that **7u** induced a mixed-type inhibition. The secondary plots (inset of Figure 3(A)) showed a good linear relationship, suggesting that the inhibitor (**7u**) bound mainly in a single kind of inhibition sites on *α*-glucosidase. The kinetic parameters (Table 2) were also obtained by the nonlinear regression of the Michaelis–Menten plots (Supplementary Figure S1) and the *K*_i_ value of this mixed-type inhibition was calculated as 2.28 μM.

**Table 2.** Kinetic parameters of *α*-glucosidase in the presence of **7u**.

Concentrations of 7u (µM)	Vmax (µM·min^−1^)	*K*_m_ (µM)	*K*_i_ (µM)
0	1.60 ± 0.03	5.19 ± 0.67	2.28 ± 0.46
1	1.39 ± 0.02	6.18 ± 0.59	
2	1.24 ± 0.01	8.17 ± 0.37	
4	1.04 ± 0.01	9.08 ± 0.53	
8	0.84 ± 0.02	10.83 ± 0.96	

#### 3.2.5. Fluorescence quenching mechanism and binding characterisations

The endogenous fluorescence property of *α*-glucosidase is mainly derived from aromatic amino acid residues, such as tryptophan and tyrosine^3^. So the fluorescence quenching experiments could be used to characterise the binding constant (*K*_a_), binding site (*n*) and mechanism of binding between **7u** and the enzyme. As shown in Figure 3(B)–(D), α-glucosidase possessed a strong fluorescence-emission peak at 350 nm, which quenched regularly with the addition of increasing the sample (**7u**) concentrations. The data suggested the change of *α*-glucosidase structure, at the meantime, reflecting the formation of *α*-glucosidase–**7u** complex.

To shed light on the interaction mechanism, the Stern–Volmer equation (Equations S5) was used to analyze the fluorescence data. The Stern–Volmer plots (the inset in Figure 3(B)) suggested that the quenching course was a single mode. Generally, there are two types of quenching i.e. dynamic quenching and static quenching. Usually, static quenching is characterised by decreasing quenching constant with increasing temperature^29^. As listed in Table 3, the values of *K*_SV_ have a negative correlation with temperature, specifying a static quenching mechanism. Furthermore, the values of *K*_q_ (10^12^ L·mol^−1^·s^−1^) far exceeded than maximum diffusion collision quenching constant (2.0 × 10^10^ L·mol^−1^·s^−1^), which also suggested a static quenching process. The intrinsic binding constant *K*_a_ and the number of binding sites *n* values of *α*-glucosidase with **7u** could be calculated by the double logarithm equation (Equations S6). The Table 3 presented the values of *K*_a_ which were in the order of 10^5^ L·mol^−1^, indicating a moderate to good binding affinity of compound **7u** for the *α*-glucosidase. In addition, the values of *K*_a_ were also inversely correlated with temperature that evoked the stability of the compound–enzyme complex decreased at a higher temperature. Furthermore, the value of *n* was approximate to 1, which indicated the presence of a single binding site for understudy compound (**7u**) on *α*-glucosidase.

**Table 3.** Quenching constants (*K*_sv_), binding constants (*K*_a_) and number of binding sites (*n*) for the **7u **− *α*-glucosidase system at different temperatures.

*T* (^o^C)	*K*_SV_ (× 10^4^ L·mol^−1^)	*R*^a^	*K*_a_ (× 10^5^ L·mol^−1^)	*n*	*R*^b^
25	5.00 ± 0.04	0.9937	4.35 ± 0.05	1.23	0.9986
31	4.74 ± 0.03	0.9905	2.50 ± 0.04	1.19	0.9990
37	4.31 ± 0.02	0.9964	1.33 ± 0.03	1.15	0.9993

^a^The correlation coefficient for the *K*_SV_ value.

^b^The correlation coefficient for the *K*_a_ value.

To further characterise the intermolecular forces between *α*-glucosidase and **7u**, the thermodynamic parameters were also calculated by using van’t Hoff equation (Equation S7 and S8), which determined the main forces contributing to the ligand–protein stability.

The values of Δ*H*° and Δ*S*° were obtained from the linearly fitted plot of log*K*_a_ against 1/*T*. The values of *K*_a_ rose with the temperature enhancement, which directed that the binding ability of **7u** to *α*-glucosidase became stronger with increasing temperature, while the binding reaction was exothermic. The negative values of Δ*G*° showed that the binding process was thermodynamically favorable and occurred spontaneously. As the calculated parameters listed in Table 4, the negative values of Δ*H*° (–75.82 kJ·mol^−1^) and Δ*S*° (–146.32 J·mol^−1^·K^−1^) indicated the action of van der Waals force and hydrogen bonds offered the dominant contribution not only in formation but also in stabilisation of the *α*-glucosidase–**7u** complex. The reaction also characterised an enthalpy-driven process due to |Δ*H*°| > |*T*Δ*S*°|.

**Table 4.** Thermodynamic parameters for the **7u **− *α*-glucosidase system.

*T* (^o^C)	Δ*G*^o^ (kJ·mol^−1^)	Δ*H*^o^ (kJ·mol^−1^)	Δ*S*^o^ (J·mol^−1^·K^−1^)
25	−32.21 ± 0.06	−75.82 ± 0.11	−146.32 ± 0.02
31	−31.33 ± 0.03		
37	−30.46 ± 0.02		

#### 3.2.6. Isothermal titration calorimetry binding assays

**Figure 4.**
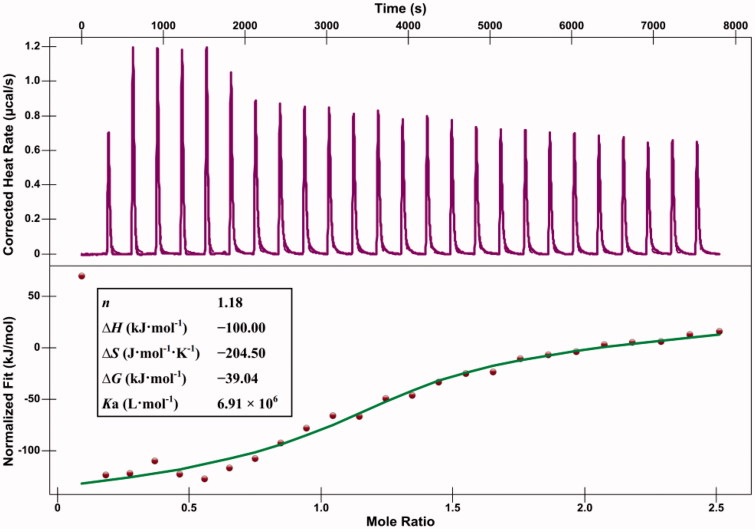
Calorimetric titration of the *α*-glucosidase with compound **7 u** at 25 °C. Heat flow as a function of time (purple). The green curve corresponds to the theoretical independent model. The thermodynamic constants are presented in the pane.

Isothermal titration calorimetry (ITC) was carried out to study the interactions between compound **7u** and *α*-glucosidase, which could give direct measurement of the binding constant, the stoichiometry, the heat of reaction, as well as the indirect access to other thermodynamic parameters such as entropic binding contribution and Gibbs free energy. As shown in Figure 4, the green curve corresponded to a binding model with a 1:1 stoichiometry, which also fitted with the change of enthalpy (Δ*H*, −100.00 kJ·mol^−1^), entropy (Δ*S*, –204.50 J·mol^−1^·K^−1^) and free energy (Δ*G*, –39.04 kJ·mol^−1^). These data indicated that the binding was enthalpy-driven and spontaneous reaction which conformed to the fluorescence result. The value of binding constant *K*_a_ was 6.91 × 10^6^ L·mol^−1^, demonstrating a good binding affinity of compound **7u** for *α*-glucosidase. Fluorescence technology and ITC are common methods for studying the interactions between small molecular ligands and protein^35^^,^^37^^,^^38^. In this work, the thermodynamic parameters obtained by these two methods showed the same trend. However, there are some difference in their values, which could be attributed to factors such as the tested solvent environment, the macromolecular state, and the electrostatic repulsions between the molecules that bear the same charge^35^. Similar situations have also been reported in the literatures^35^^,^^37^. The accurate thermodynamic parameters need to be further studied in combination with other techniques, such as surface plasmon resonance (SPR)^38^^,^^39^ and thermal shift assays (TSA)^40^^,^^41^.

#### 3.2.7. Synchronous fluorescence

**Figure 5.**
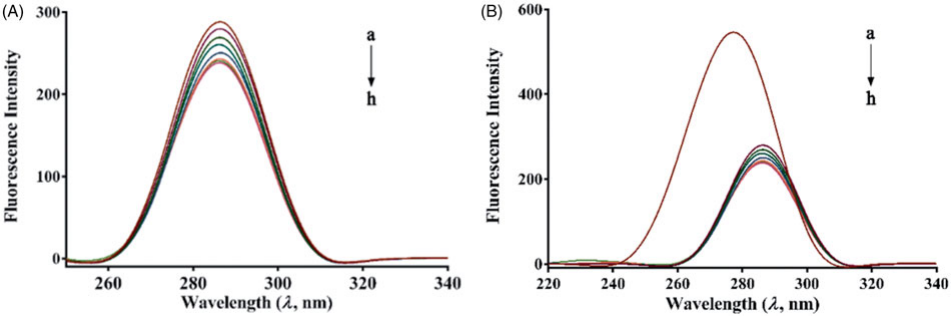
Synchronous fluorescence spectra of *α*-glucosidase in the presence of increasing concentrations of **7 u** (A) Δ*λ* = 15 nm, (B) Δ*λ* = 60 nm (pH 6.8, *T* = 25 °C). c(*α*-glucosidase) = 2 µM. c(**7u**) = 0, 0.5, 1, 2, 3, 4, 5 and 6 µM for curves a → h, respectively.

The microenvironment changes of fluorophores in *α*-glucosidase were monitored by synchronous fluorescence spectroscopy, which was done by measuring the possible shift of spectra in maximum emission wavelength before and after binding with ligand. By setting the Δ*λ* (Δ*λ* = *λ*em – *λ*ex) at 15 or 60 nm, the information about the microenvironment changes of Tyr and Trp residues were obtained, respectively^29^. As shown in Figure 5, the synchronous fluorescence intensity of both Tyr and Trp residues decreased with the concentration of **7u** increased. No evident shift was observed at the maximum emission wavelength of Tyr residues (Figure 5(A)). However, Trp residues had a noticeable red shift (from 277.5 to 286.5 nm) as shown in Figure 5(B). The result indicated that **7u** did not alter the polarity around Tyr residues significantly, while the polarity around the Trp residues enhanced and the hydrophobicity decreased. In addition, the gradually declined fluorescence intensity with increased concentration of **7u** further supported the occurrence of fluorescence quenching.

#### 3.2.8. Circular dichroism spectroscopy

**Figure 6.**
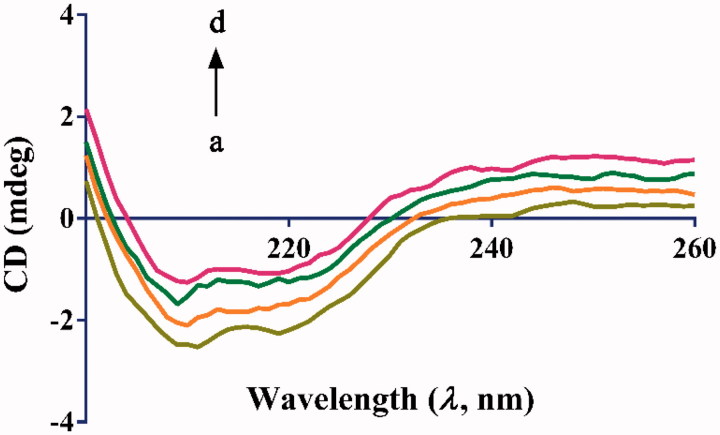
CD spectra of *α*-glucosidase in the presence of **7 u**. c(*α*-glucosidase) = 2 µM. The molar ratios of **7 u** to *α*-glucosidase were 0:1, 1:1, 2:1 and 4:1 for curves a → d, respectively.

The CD spectroscopy has considered as a reliable and sensitive method for monitoring the structural changes of bio-macromolecules in interaction with small molecules. As shown in Figure 6, the CD spectra of *α*-glucosidase exhibited a pair of negative bands at 209 and 220 nm, which were the characteristic of the *α*-helical structure of protein, originated from the *n*→π* and *π*→π* electron transfer for the peptides bonds of the *α*-helix^3^^,^^42^. On adding various concentrations of ligand (**7u**), both intensity of the negative bands decreased without any significant changes in the peak position and shape. The results revealed that binding of **7u** to *α*-glucosidase encouraged the structural transformation of polypeptides, resulting in the inhibition of *α*-glucosidase activity.

## 4. Conclusions

This work demonstrated the *α*-glucosidase inhibitory potential of natural 2,4-dihydroxy-5-methylacetophenone scaffold and its derivatives. The *in vitro* study revealed that compounds **7d**, **7f**, **7i**, **7n**, **7o**, **7r**, **7s**, **7u,** and **7v** were efficient *α*-glucosidase inhibitors with IC_50_ values of 1.68–7.88 μM, which were much stronger than that of acarbose and genistein. The most promising derivative **7u** effectively reduced the level of postprandial blood glucose *in vivo* mainly through inhibition of the activity of *α*-glucosidase. Furthermore, this research displayed that compound **7u** inhibited the activity of *α*-glucosidase in a mixed-type manner, with its *K*_i_ value of 2.28 µM. As an enthalpy-driven spontaneous process, the compound **7u** bound to *α*-glucosidase to form a complex with one affinity binding site. Overall, this research could enrich the types of candidate *α*-glucosidase inhibitors and provide more options for efficient chemotherapies in the treatment of Type-II diabetes.
